# Nationwide Seroprevalence of SARS-CoV-2 IgG Antibodies among Four Groups of Primary Health-Care Workers and Their Household Contacts 6 Months after the Initiation of the COVID-19 Vaccination Campaign in France: SeroPRIM Study Protocol

**DOI:** 10.3390/pathogens10070911

**Published:** 2021-07-20

**Authors:** Marie Pouquet, Dorine Decarreaux, Pol Prévot-Monsacré, Corentin Hervé, Andréas Werner, Brigitte Grosgogeat, Hélène Blanché, Pascaline Rabiega, Julien Laupie, Fabienne Kochert, Nathalie Abraham, Jean-Marc Sebaoun, Xavier de Lamballerie, Remi Charrel, Cecile Souty, Ibrahima Camara, Jeanne Pergeline, Harold Noël, Caroline Guerrisi, Sylvie van der Werf, Fabrice Carrat, Thomas Hanslik, Thierry Blanchon, Alessandra Falchi

**Affiliations:** 1 INSERM, Institut Pierre Louis d’Epidémiologie et de Santé Publique, (IPLESP), Sorbonne Université, 75012 Paris, France; DECARREAUX_D@univ-corse.fr (D.D.); pol.monsacre@iplesp.upmc.fr (P.P.-M.); corentin.herve@iplesp.upmc.fr (C.H.); cecile.souty@iplesp.upmc.fr (C.S.); ibrahima.camara@iplesp.upmc.fr (I.C.); jeanne.pergeline@iplesp.upmc.fr (J.P.); caroline.guerrisi@iplesp.upmc.fr (C.G.); fabrice.carrat@iplesp.upmc.fr (F.C.); thomas.hanslik@aphp.fr (T.H.); thierry.blanchon@iplesp.upmc.fr (T.B.); 2 Laboratoire de Virologie, Université de Corse Pascal Paoli, UR7310 BioScope, 20250 Corte, France; 3 Unité des Virus Emergents, Aix Marseille University, IRD 190, INSERM U1207, 13005 Marseille, France; xavier.de-lamballerie@univ-amu.fr (X.d.L.); remi.charrel@univ-amu.fr (R.C.); 4 Association Française de Pédiatrie Ambulatoire (AFPA), 69000 Orléans, France; docteur.werner.pediatre@wanadoo.fr (A.W.); fabienne.kochert@wanadoo.fr (F.K.); 5 Faculté d’Odontologie, Université Claude Bernard Lyon 1, Université de Lyon, 69000 Lyon, France; brigitte.grosgogeat@univ-lyon1.fr; 6 Laboratoire des Multimatériaux et Interfaces, UMR CNRS 5615, Université Claude Bernard Lyon 1, Université de Lyon, 69000 Lyon, France; 7 Réseau ReCOL, Association Dentaire Française, 75000 Paris, France; Reseau.recol@gmail.com; 8 Fondation Jean Dausset-CEPH, 75000 Paris, France; helene.blanche@fjd-ceph.org (H.B.); jean-marc.sebaoun@fjd-ceph.org (J.-M.S.); 9 IQVIA, Réseau de Pharmaciens, 75000 Paris, France; pascaline.rabiega@iqvia.com (P.R.); nabraham@fr.imshealth.com (N.A.); 10 Infectious Diseases Division, Santé Publique France, 94410 Saint Maurice, France; harold.noel@santepubliquefrance.fr; 11 Unit of Molecular Genetics of RNA Viruses, UMR 3569 CNRS, Institut Pasteur, University of Paris-Diderot, 75000 Paris, France; sylvie.van-der-werf@pasteur.fr; 12 Institut Pasteur, Centre Coordonnateur du Centre National de Référence des Virus des Infections Respiratoires (Dont la Grippe), 75015 Paris, France; 13 Faculty of Health Sciences Simone Veil, Université de Versailles Saint-Quentin-en-Yvelines (UVSQ), UFR de Médecine, 78000 Versailles, France; 14 Service de Médecine Interne, Hôpital Ambroise Paré, Assistance Publique—Hôpitaux de Paris (APHP), 92100 Boulogne Billancourt, France

**Keywords:** SARS-CoV-2, seroprevalence, vaccination, primary health-care workers, household

## Abstract

Background: The protocol study will focus on the seroprevalence of IgG antibodies to SARS-CoV-2 achieved by vaccination and/or natural protection as well as the history, symptoms, and risk factors for SARS-CoV-2 in four primary health-care workers (PHCWs) and their household contacts in metropolitan France. Methods: Here, we propose a protocol for a nationwide survey to determine the seroprevalence of IgG antibodies to SARS-CoV-2 achieved by vaccination and/or natural protection in four PHCW populations (general practitioners, pediatricians, pharmacists and assistants, and dentists and assistants) and their household contacts. Participants will be included from June to July 2021 (Phase 1) among PHCW populations located throughout metropolitan France. They will be asked to provide a range of demographic and behavioral information since the first SARS-CoV-2 wave and a self-sampled dried blood spot. Phase 1 will involve also a questionnaire and serological study of PHCWs’ household contacts. Seroprevalence will be estimated using two ELISAs designed to detect specific IgG antibodies to SARS-CoV-2 in humoral fluid, and these results will be confirmed using a virus neutralization test. This study will be repeated from November to December 2021 (Phase 2) to evaluate the evolution of immune status achieved by vaccination and/or natural protection of PHCWs and to describe the history of exposure to SARS-CoV-2.

## 1. Introduction

The ongoing coronavirus disease 2019 (COVID-19) pandemic caused by the severe acute respiratory syndrome coronavirus 2 (SARS-CoV-2) represents a significant burden for health services and health-care workers (HCWs) [1,2]. HCWs can be exposed to SARS-CoV-2 in the clinical setting as well as at home and in social situations [3]. A recent meta-analysis of 49 studies estimated an overall seroprevalence of SARS-CoV-2 antibodies IgG acquired by natural immunity among HCWs of 8.7% (95% confidence interval 6.7–10.9%) [3]. In this context, priority has been given to the vaccination of HCWs to maintain the provision of critical services and to reduce the spread of infection in health-care settings and in people at risk of COVID-19 complications and death.

Vaccination against COVID-19 began worldwide at the end of 2020 with the use of RNA-based vaccines using epitopes of the SARS-CoV-2 spike (S) protein to induce protective immunity [4]. Since the introduction of vaccines against SARS-CoV-2, data on vaccine effectiveness among HCWs in the field are beginning to report notable reductions in the rate of SARS-CoV-2 infection among vaccinated compared with unvaccinated HCWs to less than 1 case per 1000 HCWs tested [5]. The first US multisite test-negative design vaccine effectiveness study among HCWs found a single dose of an mRNA vaccine (Pfizer-BioNTech or Moderna COVID-19) to be 82% effective against symptomatic COVID-19, and two doses to be 94% effective [6]. The detection of neutralizing and IgG antibodies in 96.5% and 99.9% of vaccinated HCWs in an Israeli study suggests that the Pfizer-BioNTech BNT162b2 COVID-19 vaccine elicits a significantly higher and more robust antibody response compared to natural infection [7]. Vaccination of HCWs has also been associated with a substantial reduction in the number of COVID-19 cases among household contacts, which is consistent with the effectiveness of vaccination for preventing transmission [8]. Although neither of the studies [6,7] specifically assessed the rates of asymptomatic SARS-CoV-2 infection, recent studies suggest that asymptomatic SARS-CoV-2 infection may be frequent in vaccinated frail older patients [9] and less frequent, yet observable, in the general population [10]. These results support the use of vaccines in addressing the COVID-19 pandemic, but it is not clear whether the immune protection will last or whether vaccine boosters will be required to provide long-lasting protection. However, several cases of “vaccine breakthrough cases” with SARS-CoV-2 variants inoculated with one or both doses of vaccine have been reported [11], suggesting that a relatively modest percentage of fully vaccinated individuals may become infected when exposed to the virus. In Italy, the first case of SARS-CoV-2 P.1.1 infection lacking N501 mutation in a fully vaccinated (Pfizer) 22-year-old female nurse working in the COVID center has been reported [12]. 

In the era of SARS-CoV-2 vaccination, seroprevalence studies to monitor recent natural infection are essential in understanding and evaluating the global performance of vaccines, especially in at-risk populations such as HCWs. Understanding infection rates in HCW vaccinated populations is important because HCWs are at an increased risk of SARS-CoV-2 infection [13] and, when infected, they also present a risk of transmission to other HCWs and uninfected patients. Uninfected but vaccinated people should develop measurable levels of antibodies as assessed in serological assays targeting the SARS-CoV-2 S protein but not in assays targeting the nucleocapsid (N) protein of SARS-CoV-2 [14]. It has been suggested that IgG antibodies targeting the S protein are more specific, whereas those targeting the N protein may be more sensitive, particularly in the early phase of infection [14]. Antibody responses against viral S and N proteins are equally sensitive during the acute phase of infection, but SARS-CoV-2 anti-nucleocapsid antibody titers wane within months, and faster in younger adults and those without symptoms, whereas those against the S protein persist over time [15].

Therefore, it should be possible to differentiate between a vaccination response and a recent natural infection with SARS-CoV-2 based on these different test strategies. Consequently, antibody tests that target both the S and N proteins may be able to identify prior recent infection among vaccinated people [14].

Most reports describing the burden of SARS-CoV-2 infection among HCWs have focused on hospital HCWs. Since the beginning of the SARS-CoV-2 pandemic in early 2020, primary care physicians and caregivers in the ambulatory sector have adapted to the growing challenges posed by COVID-19. General practitioners and pediatricians have organized, in addition to teleconsultations, dedicated circuits and specific care for suspected COVID-19 patients. As a vital part of the health-care system, pharmacists have worked continuously during the COVID-19 pandemic by remaining at the front line of public health and serving as a direct point of access for their patients. Because saliva is the main means of SARS-CoV-2 spread, dentistry is one of the medical practices at the highest risk of infection given the frequent production of aerosols and the constant presence of saliva during dental procedures.

## 2. Materials and Methods

### 2.1. Study Design

Here, we propose a nationwide longitudinal survey to determine the seroprevalence of IgG antibodies to SARS-CoV-2 achieved by vaccination and/or natural protection in four primary HCW populations (PHCWs: general practitioners [GPs], pediatricians, pharmacists and assistants, and dentists and assistants) and its temporal evolution. We will also conduct a cross-sectional study to investigate antibodies to SARS-CoV-2 among their household contacts. Participants will be included from June to July 2021 (Phase 1: baseline survey) among PHCW populations located throughout metropolitan France. They will be asked to provide a range of demographic and behavioral information since the first SARS-CoV-2 wave and a self-sampled dried blood spot. This phase will involve also a questionnaire and serological study of PHCWs’ household contacts since the first SARS-CoV-2 wave. Seroprevalence will be estimated using two ELISA-designed assays to detect specific IgG antibodies against viral S and N proteins of SARS-CoV-2 in humoral fluid, and these results will be confirmed using a virus neutralization test (VNT). The VNT will also evaluate the levels of functional neutralizing SARS-CoV-2 antibodies. This step will allow evaluation of the immune response rates to SARS-CoV-2, the impact of COVID-19 vaccine uptake on these rates, and prevalence and risk factors of SARS-CoV-2 infection. This study will be repeated from November to December 2021 (Phase 2) to evaluate the evolution of immune status achieved by vaccination and/or natural protection of PHCWs and to describe the history of exposure to SARS-CoV-2. This study was registered at https://www.clinicaltrials.gov on 17 March 2021. The study protocol has been written in accordance with the recommendations outlined in the Strengthening the Reporting of Observational Studies in Epidemiology (STROBE) checklist.

### 2.2. Objectives

The protocol study will focus on the seroprevalence of IgG antibodies to SARS-CoV-2 achieved by vaccination and/or natural infection as well as the history, symptoms, and risk factors for SARS-CoV-2 in four PHCWs and their household contacts in metropolitan France. Identifying the extent of SARS-CoV-2 vaccination, spread of variants, and risk factors for SARS-CoV-2 infection especially among vaccinated PHCWs will allow public health authorities to adjust prevention recommendations and target those factors that contribute the most to the protection of PHCWs and their household contacts.

The following study questions will be addressed.

Phase 1 (baseline survey of PHCWs and enrollment of their household contacts)

- What is the SARS-CoV-2 antibody prevalence achieved by vaccination and/or natural infection among the four PHCW populations and each PHCW subpopulation?
- What proportion of vaccinated PHCWs and household contacts develops SARS-CoV-2 infection?
- What are the risk factors for SARS-CoV-2 infection for PHCWs and household contacts?
- Is the risk of SARS-CoV-2 infection of PHCW household members higher than that observed in the general population?

Phase 2 (follow-up of PHCWs)

- Is there a change in antibody titers achieved by vaccination and/or natural infection of PHCWs who tested positive by serological analysis in Phase 1?
- What proportion of vaccinated PHCWs develops a new SARS-CoV-2 infection?

The specific aims are as follows:

- to estimate repeatedly the seroprevalence of IgG against the SARS-CoV-2 S protein (ELISA-S), N protein (ELISA-N), and neutralizing antibodies in the four PHCW populations in the first baseline phase in June–July 2021 and 6 months after enrollment in November–December 2021 (~6 months and ~12 months after the initiation of the COVID-19 vaccination campaign, respectively)
- to estimate repeatedly the level of S protein-binding IgG and neutralizing antibodies in the vaccinated groups with and without previous documented natural exposure in June–July 2021 and November–December 2021
- to determine the antibody persistence acquired by natural exposure and/or vaccination
- to identify factors, including sociodemographic characteristics, health history, vaccination status, and occupational behaviors, associated with the antibody response to SARS-CoV-2 in PHCWs and household contacts.

### 2.3. Recruitment

#### 2.3.1. Phase 1: PHCWs and Household Members

PHCWs will be recruited using the following four networks: (1) the French Sentinelles Network, which collects real-time epidemiological data from 1338 GPs for surveillance and research purposes [12]; (2) IQVIA, an international company that collects data from 14,000 records of retail pharmacies and primary care electronic medical records; (3) the French Association of Ambulatory Pediatrics (AFPA), a nonprofit association with 1500 pediatricians that aims to promote medical research in the field of ambulatory pediatrics; (4) Clinical Research in Liberal Dentistry (RECOL), a national research network with 830 liberal dentists, and aims to conduct studies that integrate the daily clinical activity of French dentists. This recruitment process will allow us to rapidly initiate and collect data of over 2000 PHCWs and to ensure that the study is properly designed according to professional specialty.

Various approaches for recruitment will be used, including e-mail communication sent by each network, information about the study at virtual meetings, and mentions in the networks’ social media. PHCW inscription began in May 2021 and will close in mid-June 2021.

All households of PHCWs enrolled in Phase 1 will be invited to join the study.

#### 2.3.2. Phase 2: PHCWs Follow-Up

All PHCWs whose serological status is defined at enrollment and who completed the questionnaire will be invited to participate again.

### 2.4. Eligibility Criteria

#### 2.4.1. Inclusion Criteria for PHCWs

To be included in this study, participants will need to fulfill the following inclusion criteria:

- being a PHCW in a primary care setting (GPs, pharmacists, dentists or assistants, pediatricians or assistants) in metropolitan France at the time of recruitment
- being affiliated with the French health insurance system.

#### 2.4.2. Inclusion Criteria for Household Contacts

- PHCW has agreed to the household investigation
- individuals living at least 2 days a week with a PHCW included in this study
- household contacts of any age.

#### 2.4.3. Exclusion Criteria (PHCWs and Households Contacts)

- having participated in a chemoprophylaxis clinical trial of SARS-CoV-2 infection
- being subjected to legal protection measures.

### 2.5. Participant Pathway

**Figure 1:**
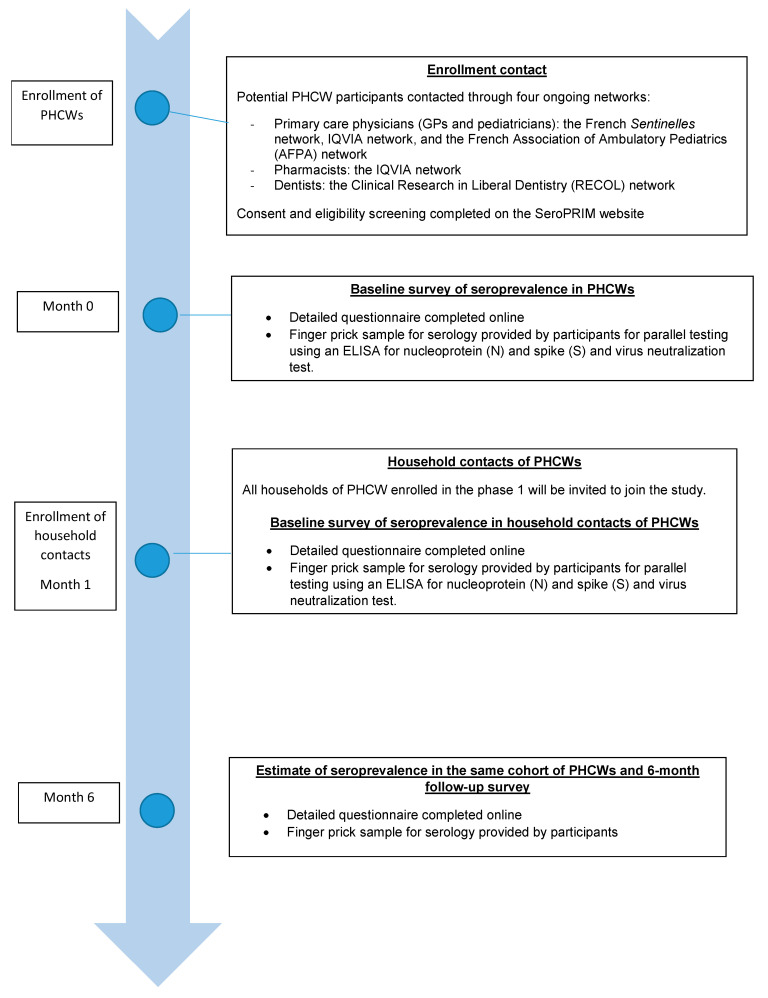
Participant pathway.

This section provides the pathway details of study participation from enrollment to the end of the study (Figure 1).

### 2.6. Study Procedure

#### 2.6.1. PHCWs Consent Procedure

PHCWs will be recruited by e-mail communication from the networks mentioned above. During the two phases, potential participants will be given a brief explanation of the study and will choose to participate by following the link in the recruitment materials to a survey on the study website (SeroPRIM website). Using this platform, before the data collection, participants will complete a prescreening questionnaire and an electronic informed consent form before being deemed to be eligible. In Phase 2, PHCWs who participated in Phase 1 will be asked by e-mail to participate in the second phase of the study.

#### 2.6.2. Household Contacts

If the PHCW agrees to the household investigation in the baseline survey (Phase 1), the study team will contact their household members about study participation by e-mail. Before data collection, household members who agree to participate will be asked to sign the consent form electronically on the study website (SeroPRIM website).

#### 2.6.3. Data Collection

In Phases 1 and 2, data collection will include the analysis of capillary blood collected on a dried blood spot (DBS) and an online questionnaire for PHCWs and household contacts. PHCWs and household contacts will receive at the mailing address they gave on the study website a self-sampling DBS kit that includes a DBS card, lancets, a pad, detailed printed instructions about performing the blood sampling, and a self-addressed stamped padded envelope to return the DBS card to the centralized biobank (CEPH Biobank, Paris, France). When they receive the DBS kit, participants will be invited to perform the blood sampling and complete an online questionnaire at the same time. Successful participation in the study will be defined as providing both the blood sample for serological analysis and a completed questionnaire.

In Phase 1, the online questionnaire for PHCWs will include questions about age, sex, household size and composition, comorbidities, tobacco use, immunosuppressive therapy, known contact with people with COVID-19, results of any diagnostic testing such as molecular testing/antibody testing for COVID-19, a detailed description of the subject’s symptoms after COVID-19 diagnosis, and history of vaccination against SARS-CoV-2. PHCWs will be asked to provide a detailed description of occupational exposure to SARS-CoV-2 and of practices since the beginning of the pandemic. Each household member will be asked to complete the survey with questions about demographics, occupation, medical history, clinical symptoms, SARS-CoV-2 exposure, and past SARS-CoV-2 testing.

In Phase 2, the online questionnaire for PHCWs will include questions about the results of any diagnostic testing, such as molecular testing/antibody testing for COVID-19, a detailed description of the PHCW’s symptoms after COVID-19 diagnosis, and history of vaccination against SARS-CoV-2 since enrollment in Phase 1. Further, PHCWs will be asked to provide a detailed description of occupational exposure to SARS-CoV-2.

#### 2.6.4. Data Management

All participants will be assigned a participant number based on their order of inclusion. Collected data from serological tests and questionnaires will be de-identified, and databases will be compiled using the unique participant ID. Only de-identified nonpersonal data will be shared among the research team for statistical analysis. Personal and nominative data, including e-mail and postal addresses, will be used exclusively for follow-up purposes and sending the DBS kit, and this information will be kept in an electronic database that will be encrypted and password protected, and access will be restricted to the study coordinator and three people responsible for the study coordination. The personal and nominative data will be stored electronically separately from the coded, de-identified research data.

#### 2.6.5. Blood Preparation and Serological Measurements

The blood spots in the returned DBS kits will be assessed visually and registered in the CEPH Biobank LIMS (BIOBASE). Four discs will be punched from the spots using a Panthera™ device (PerkinElmer) and stored at room temperature in 2D barcoded tubes. Tubes will be sent to the virology laboratory at the Unité des virus Émergents, Marseille, France, for serological analysis. DBS kits are stored in a freezer at −30 °C.

In order to discriminate between a vaccination response and a recent natural infection, serum samples will be analyzed by using ELISA assays targeting the S and N proteins of SARS-CoV-2, as uninfected but vaccinated participants develop measurable levels of antibodies against the protein S but not in assays targeting the protein N. All samples will be analyzed using an ELISA to detect anti-SARS-CoV-2 IgG directed against the S1 domain of the spike protein of the virus (Euroimmun^®^, Lübeck, Germany, ELISA-S). All samples with an ELISA-S test optical density ratio ≥ 0.7 will be analyzed for the SARS-CoV-2 N protein (Euroimmun^®^, Lübeck, Germany, ELISA-N). All samples, regardless of the result of the ELISA-S assay, will be analyzed with an in-house microneutralization assay to detect neutralizing anti-SARS-CoV-2 antibodies [16]. The neutralization titer will be defined as the highest dilution of positive serum still demonstrating neutralization. VeroE6 cells cultured in 96-well microplates, 100 fifty-percent tissue culture infective doses (TCID_50_) of the SARS-CoV-2 strain BavPat1 (courtesy of Pr. Drosten, Berlin), and serial dilutions of serum (1/20–1/160) will be used. Dilutions associated with cytopathic effect (CPE) will be considered negative (no neutralization) and those with no CPE at day 4 post-infection will be considered positive (complete neutralization). The neutralization titer refers to the highest dilution of serum with a positive result. Specimens with a VNT titer ≥ 40 were considered positive in agreement with previous studies [17,18].

#### 2.6.6. Data Analysis

##### Outcomes

- The main outcome measure is the prevalence of SARS-CoV-2 infection, defined as either development of SARS-CoV-2 antibodies, as determined by ELISA-S and ELISA-N, and/or self-declared SARS-CoV-2 infection that has been biologically confirmed
- The secondary outcome measure is a positive VNT defined as a titer ≥ 40.

We will classify infected people on the basis of the presence or absence of obvious symptoms as reported in the questionnaire and on the basis of ELISA and seroneutralization results. Serum samples will be evaluated for antibodies against the SARS-CoV-2 N, enabling differentiation between vaccination responses and natural infection, in the limits of the sensibility and specificity of the ELISA test. Thus, we will be able to classify people not declaring symptoms but with antibodies against the SARS-CoV-2 N as asymptomatic.

##### Sample Size

The sample size for the PHCW population was calculated assuming an a priori 10% seroprevalence of anti-SARS-CoV-2 IgG acquired by natural immunity (ELISA-S+ and ELISA-N+) among participants in each PHCW subpopulation, in accordance with the literature [3,17], a confidence in the estimate of 95% [19], and a maximum allowable error in the prevalence of 3%. 

Allowing for a minimum 20% dropout rate, we estimate that, for each subpopulation, we will need to recruit about 500 PHCWs. With four subpopulations, we anticipate recruiting about 2000 PHCWs. To ensure the calculation of the rate of seroprevalence after adjustment for age, sex, and region, particular attention will be paid during recruitment to ensure an adequate sample size in each of the following strata predefined according to age (<40, 40–59, ≥60 years), sex (men and women), and region (the five following regions: South-West, South-East, North-West, North-East, and Île de France). All households of participating PHCWs in the study will be included. 

##### Proposed Statistical Analyses

Crude and standardized (age, sex, and region) seroprevalence of IgG against the SARS-CoV-2 spike protein (ELISA-S) and nucleocapsid protein (ELISA-N) and neutralizing antibodies and their 95% confidence intervals will be estimated in the first baseline phase (PHCWs and household members) and in the second phase (PHCWs). Weights will be assigned to each participant based on their membership to each of predefined strata of age–sex–region. We will define post-stratification weights as the proportion of each stratum represented in the four PHCW French populations (for PHCWs) and in the general population (for household contacts) divided by the analogous proportion in the sample for the four PHCW populations and their household contacts. Infection rate among vaccinated PHCWs will also be estimated by dividing the number of acquired infections—defined as either development of SARS-CoV-2 antibodies, as determined by ELISA-S and ELISA-N, and/or self-declared SARS-CoV-2 infection that has been biologically confirmed—by the total number of following PHCWs during the follow-up period. We will also calculate an “adjusted seroprevalence”, taking into account the specificity and the sensibility of the diagnostic tests [20].

The study population will be described in terms of demographic characteristics (age, sex, region) and occupation. Dichotomous variables will be used to compare using the chi-square test and Fisher’s exact test, and continuous variables will be analyzed using a t test or Mann–Whitney U test depending on the distribution. A series of analyses will be conducted using logistic regression models to identify the factors associated with seropositivity. Multiple logistic regressions with backward elimination will be used to identify independent factors associated with SARS-CoV-2 seropositivity or ELISA-S+. Factors associated with infection despite vaccination will also be identified. *p* < 0.05 will be considered to be significant.

### 2.7. Ethics and Dissemination

#### 2.7.1. Informed Consent

The informed consent form of the study contains information about the study objectives and process, potential risks and benefits, and the use and storage of data and biological specimens for this study and possible further research on respiratory viruses. Participants are provided with a link to a website containing further information about the study. The forms have been reviewed by the ethics committee that authorized the trial.

Electronic informed consent will be obtained before inclusion from all participants or their legal guardians and will include consent for children. The participant/legal guardian/child will be free to decline or withdraw consent at any time without providing a reason and without being subject to any resulting detriment. Children who turn 18 years old between the time of consent and the data collection period will be invited to provide their consent for the study. Participants will be informed of the results if they indicate this request in their consent form. Additional consent will be sought to store anonymized specimens and collected data for future research on respiratory viruses.

#### 2.7.2. Data Protection

Collected data will be stored securely at the Institut Pierre Louis d’Epidémiologie et de Santé Publique (IPLESP), Paris, France. De-identified data for participants who consent to the use of their data for further research may be made available to other researchers beyond the protocol stipulations without additional ethics approval. Personal and nominative data will be kept in an electronic database, which will be encrypted and password protected, and access restricted to the study coordinator and three other people responsible for the study coordination. Under no circumstances will these data be made available to a third party. The personal and nominative data will be electronically stored separately from the coded de-identified research data. De-identified data will be destroyed after 20 years.

#### 2.7.3. Dissemination

The findings of the study will be disseminated as peer-reviewed publications in journals. They will be reported in accordance with the recommendations outlined in the STROBE checklist. According to the information sheet, participants who request to be informed of the results in their consent forms will be sent the overall results at the end of the study.

## 3. Discussion

The SeroPRIM-COVID study is the first French nationwide seroprevalence study that will report on the impact of SARS-CoV-2 infection among PHCWs and their household contacts after SARS-CoV-2 vaccination. Important data about the impact of vaccination on seroprevalence rates will also be reported.

### 3.1. Strengths of the Study

The present study will enroll a large prospective cohort of four subpopulations of PHCWs within the same geographical area (metropolitan France) and timeframe. This will mean that the seroprevalence estimates will be comparable between the studied populations as well as with the results of the SAPRIS study [17], which is using identical serological methods and is being conducted concomitantly among the general population in metropolitan France. The geographic spread of PHCW subpopulations across all of metropolitan France will provide details about regional variations in seroprevalence and data useful to the national vaccination strategy. The prospective approach of this study will provide an estimate of the infection rate and the identification of SARS-CoV-2 risk factors especially among vaccinated PHCWs. This cohort of PHCWs and their household members provide an ideal opportunity to address the question of the effects of vaccination on transmission.

### 3.2. Limitations of the Study

This study has several limitations. First, even though efforts will be made to adjust the seroprevalence estimate for age, sex, and geographical area, the convenience sampling strategy may introduce selection bias. This study may attract known recently infected people and their household contacts to join to check their infection status, or already vaccinated participants or those not yet vaccinated who have been in contact with a person who tested positive for COVID-19 while not wearing personal protective equipment. Known infected and immune people may be less motivated to join the study considering that they already know their serological status. The impact of potential selection bias on the estimation of the seroprevalence is not known and may be of different origin.

Second, the serological tests that will be used are imperfect tests, which means that some participants will probably be misclassified. We will adjust the seroprevalence data for the antibody test characteristics (sensitivity and specificity). However, we cannot be certain that misclassification will not occur using our calculation of true prevalence based on the serological status of PHCWs and their history of COVID-19 diagnosis and seropositivity of PHCWs. 

Third, recall bias should also be considered, as participants may not be able to remember the mild symptoms they had at the time of infection, leading to misclassification between symptomatic and asymptomatic.

## 4. Conclusions

Despite these limitations, our study will be of a high quality compared with the seroprevalence studies conducted to date. A recent meta-analysis reported that the overall quality of existing seroprevalence studies of SARS-CoV-2 is low and that these studies include sampling strategies that are not rigorous, use poorly validated and unstandardized laboratory methods, and lack statistical correction for demographics and test performance in their analyses [21]. Classifying our study according to the quality scoring system described in the meta-analysis of Chen et al. [21] indicates that our study would be classified as grade B and would be included in the top 20% of studies with the highest quality and would thus be considered as a good quality study.

## Data Availability

Not Applicable.
